# Inflammation Enhances the Risks of Stroke and Death in Chronic Chagas Disease Patients

**DOI:** 10.1371/journal.pntd.0004669

**Published:** 2016-04-26

**Authors:** Paulo Marcos Matta Guedes, Cléber Mesquita de Andrade, Daniela Ferreira Nunes, Nathalie de Sena Pereira, Tamyres Bernadete Dantas Queiroga, George Luiz Lins Machado-Coelho, Manuela Sales Lima Nascimento, Maria Adelaide Do-Valle-Matta, Antônia Cláudia Jácome da Câmara, Egler Chiari, Lúcia Maria da Cunha Galvão

**Affiliations:** 1 Department of Microbiology and Parasitology, Federal University of Rio Grande do Norte, Rio Grande do Norte, Natal, Brazil; 2 Department of Biomedical Sciences, University of Rio Grande do Norte State, Rio Grande do Norte, Mossoró, Brazil; 3 Department of Parasitology, Federal University of Minas Gerais, Minas Gerais, Belo Horizonte, Brazil; 4 School of Medicine, Federal University of Ouro Preto, Ouro Preto, Minas Gerais, Brazil; 5 Ribeirão Preto Medical School, University of São Paulo, São Paulo, Ribeirão Preto, Brazil; 6 Laboratory of Cellular Ultrastructure, Oswaldo Cruz Institute/FIOCRUZ, Rio de Janeiro, Rio de Janeiro, Brazil; 7 Department of Clinical and Toxicological Analyses, Federal University of Rio Grande do Norte, Natal, Brazil; Albert Einstein College of Medicine, UNITED STATES

## Abstract

Ischemic strokes have been implicated as a cause of death in Chagas disease patients. Inflammation has been recognized as a key component in all ischemic processes, including the intravascular events triggered by vessel interruption, brain damage and repair. In this study, we evaluated the association between inflammatory markers and the death risk (DR) and stroke risk (SR) of patients with different clinical forms of chronic Chagas disease. The mRNA expression levels of cytokines, transcription factors expressed in the adaptive immune response (Th1, Th2, Th9, Th17, Th22 and regulatory T cell), and iNOS were analyzed by real-time PCR in peripheral blood mononuclear cells of chagasic patients who exhibited the indeterminate, cardiac, digestive and cardiodigestive clinical forms of the disease, and the levels of these transcripts were correlated with the DR and SR. Cardiac patients exhibited lower mRNA expression levels of GATA-3, FoxP3, AHR, IL-4, IL-9, IL-10 and IL-22 but exhibited higher expression of IFN-γ and TNF-α compared with indeterminate patients. Digestive patients showed similar levels of GATA-3, IL-4 and IL-10 than indeterminate patients. Cardiodigestive patients exhibited higher levels of TNF-α compared with indeterminate and digestive patients. Furthermore, we demonstrated that patients with high DR and SR exhibited lower GATA-3, FoxP3, and IL-10 expression and higher IFN-γ, TNF-α and iNOS mRNA expression than patients with low DR and SR. A negative correlation was observed between Foxp3 and IL-10 mRNA expression and the DR and SR. Moreover, TNF-α and iNOS expression was positively correlated with DR and SR. Our data suggest that an inflammatory imbalance in chronic Chagas disease patients is associated with a high DR and SR. This study provides a better understanding of the stroke pathobiology in the general population and might aid the development of therapeutic strategies for controlling the morbidity and mortality of Chagas disease.

## Author Summary

Chagas disease is caused by *Trypanosoma cruzi (T*. *cruzi)*, affects 5.7 million people worldwide and causes 12,000 deaths annually. In the chronic phase of Chagas disease the main cause of death is due to heart failure (about 80%), but cerebral vascular accident or stroke (about 10%) contributes to death mechanisms. Strokes are caused by the interruption of the blood supply to the brain and can be ischemic or hemorrhagic. Stroke is the leading cause of death among adults in Latin America and the second in the world. Infectious diseases, such as Chagas disease, malaria, cysticercosis, tuberculosis, brucellosis and neurosyphilis, can also contribute to the development of immunopathogenic mechanisms leading to stroke and death. In this study, we evaluated the association between inflammatory markers (cytokines, transcription factors of the adaptive immune response and iNOS) and the death risk (DR) and stroke risk (SR) of patients with different clinical forms of chronic Chagas disease. Our data suggest that an inflammatory imbalance in chronic Chagas disease patients is correlated with a high DR and SR. The exacerbated inflammatory mechanism that leads to thrombus formation can lead to sudden death in patients with clinical indeterminate form without prior other clinical symptoms. These inflammatory mechanisms are also involved in atherosclerotic-related strokes. An improved understanding of the immunological mechanisms involved in ischemic stroke formation in Chagas disease patients may also contribute to the reduction of stroke-related mortality and morbidity in the general population and may lead to the development of prophylactic or therapeutic therapies.

## Introduction

Chagas disease is caused by flagellate protozoa *Trypanosoma cruzi* (*T*. *cruzi*) and affects 5.7 million people worldwide. The disease causes morbidity in about 300,000 people disabling for work or daily living activities and causes 12,500 deaths annually [1,2]. In the chronic phase of Chagas disease, the most common cause of death is sudden cardiac death (55–65% of patients), usually due to ventricular fibrillation, followed by congestive heart failure (25–30% of patients) and pulmonary or cerebral ischemia (10–15% patients) [3]. Death from cardiac insufficiency has been reported in individuals (functional class III and IV) with reduced left ventricular ejection fraction/LVEF (<35%) [4]. In patients with reduced or preserved systolic function, ischemic stroke has often been linked as a cause of death [5,6]. Postmortem analysis of Chagas disease patients reveals brain lesions in up to 60% of cases due to ischemic stroke [7–10]. Several methods to predict the death risk in patients with chronic Chagas disease have been described based on clinical features [5,11,12]. Death and stroke are not necessarily related in Chagas disease, cardiovascular diseases involving atherosclerosis and hypertension are major causes of heart attacks and stroke in the population leading to sudden death [13,14]. However, inflammation is one of the key drivers of atherosclerotic plaque development [13]. Other established risk factors are high cholesterol, hypertension, diabetes, alcohol use, overweight, stress, smoking, sedentary lifestyle [15].

The effects of stroke depend on which part of the brain is injured and how severely it is affected; a very severe stroke can cause sudden death. In strokes caused by arterial occlusion or ischemic stroke, inflammation has been recognized as a key component of the pathophysiology of the brain [16]. Recent studies have suggested that the immune response is involved in all ischemic processes, including the intravascular events triggered by vessel interruption, brain damage and repair [17,18]. A key mediator of endothelial dysfunction is the pro-inflammatory transcription factor NF-κB. This molecule is expressed in endothelial cells and leukocytes and leads to the transcription of pro-inflammatory genes, such as cytokines, chemokines and leukocyte adhesion molecules, including vascular cell adhesion molecule-1 (VCAM-1) and E-selectin [19]. Acute immune activation after stroke is responsible for secondary brain injury [20]. After arterial occlusion, the production of reactive oxygen species (ROS) triggers the coagulation cascade and leads to the activation of complement, platelets and endothelial cells [21]. Cerebral ischemia induces the expression of TNF-α, IL-1β, IL-6 and inducible nitric oxide synthase (iNOS), which leads to the upregulation of endothelin receptors in the cerebral arteries [22,23]. The immune response generated in this context, dominated by IFN-γ and TNF-α, may facilitate vessel contraction and increase the vulnerability of the brain to cerebral ischemia [24].

Infection by *T*. *cruzi* induces a strong inflammatory response dominated by the Th1 pattern, with IFN-γ and TNF-α production and regulated by the IL-10 production [25]. The *T*. *cruzi* antigens presented by dendritic cells (DC) initiate the programmed differentiation of naïve CD4^+^ T cells into Th1 (T-Bet transcription factor; IFN-γ and TNF-α production), Th2 (GATA-3; IL-4, IL-5, IL-9, IL-10, IL-13), Th17 (RORγt and RORα; IL-17, IL-22, IL-23, IL-26, TNF-α), regulatory T cells (Treg) (Foxp3; IL-10, TGF-β, IL-35), Th9 cells (PU.1; IL-9, IL-10, IL-21) and Th22 cells (aryl hydrocarbon receptor/AHR; IL-22, TNF-α) [26–32]. These cytokines and transcriptional factors are not exclusively expressed by the subsets of CD4+ T cells (Th1, Th2, Th9, Th17, Th22, regulatory T cell). However, T-Bet, GATA3, PU.1, RORγt and FoxP3 are indispensable for Th1, Th2 [33–35], Th9 [28,36], Th17 [26,37,38] and regulatory T cell [39–42] profiles, respectively. There is no evidence of a signature marker for Th22 profile, but several literature data have been shown that aryl hydrocarbon receptor (AHR) is critical for Th22 cells [29,43,44]. The roles of Th9 and Th22 cells during Chagas disease remain unclear. Moreover, the correlations among immunological mechanisms, stroke and death have not been investigated in depth in chronic Chagas disease patients. Here, we demonstrated that indeterminate patients exhibit increased expression of Th2-, Th9-, Th22- and Treg-related cytokines and transcription factors and reduced expression of the inflammatory cytokines IFN-γ and TNF-α. In addition, patients who exhibited a high long-term death and stroke risk also exhibited increased iNOS mRNA expression, which is positively correlated with the risks of death and stroke. Together, the data indicate that uncontrolled inflammation caused by *T*. *cruzi* influences the mechanisms that lead to stroke and death during the chronic phase of Chagas disease. This knowledge may contribute to the reduction of stroke risk and death during the chronic phase of Chagas disease and may also benefit the general population.

## Methods

### Study Population

A total of 65 chagasic patients from the rural zone of Rio Grande do Norte, Brazil were selected using two different serological methods (Chagatest" recombinant ELISA and HAI, and indirect immunofluorescence assay) between 2011 and 2013. The exclusion criteria included the following: over 70 years of age, diabetes, sustained ventricular tachycardia or ventricular fibrillation, an implanted cardiac pacemaker and non-chagasic cardiomyopathy. Individuals that tested positive for Chagas disease by two serological tests with distinct testing methods underwent a complete clinical evaluation, including electrocardiogram (ECG) mapping and chest X-ray, contrasted X-rays of the esophagus and colon, 2D-echocardiogram (ECHO) and 24-h Holter examination. They were classified according to the clinical form of the disease as: cardiac, digestive or indeterminate as recommended by Brazilian Consensus on Chagas Disease [45]. Clinical evaluations were performed as described previously [46]. Following these examinations, the patients were classified as having the indeterminate (n = 18), cardiac (n = 17), digestive (n = 15) or cardiodigestive (n = 15) clinical forms of the disease. Healthy, uninfected individuals (n = 15) served as controls. Patient groups enrolled in this study did not exhibit a large number of cardiovascular risk factors. Concerning this topic, variables such as hypertension, obesity, dyslipidemia, sedentary behavior, and smoking were evaluated in study population (Table 1). The risks for stroke and death are multifactorial and depend on these factors. Thus, what determines whether patients are at higher risk for death or stroke is not exclusively an assignment of a particular cytokine, but refers to a set of factors. Multifactorial data analysis was not used in this cross-sectional study as this statistical approach is intended to modelling the massive amount of data collected from patients throughout longitudinal studies, being the resulting model usually adjusted or updated for other individuals in a process of external validation with new individuals to determine risk factors [47–49]. The present study is not aimed to propose or implement a predictive model for death and stroke risk in Chagas disease patients, but highlights the possible correlation between inflammation and these clinical manifestations.

**Table 1:** Cardiovascular risk factors of chronic chagasic subjects from the Northeast of Brazil included in this investigation.

Clinical Form				
Variable	Indeterminaten = 18	Cardiac formn = 17	Digestiven = 15	Cardiodigestiven = 15
Hypertension	11%	18%	27%	47%
	Two patients with stage-1 controlled hypertension both using angiotensin-converting-enzyme (ACE) inhibitor	Three controlled hypertensive (Stage 1 and Stage 2) patients, two using beta-blocker (BB) and angiotensin receptor blockers (ARBs) in non-optimized dose and one using ARBs.	Four hypertensive (Stage 1 and Stage 2) patients, two being controlled by changes in lifestyle, and two with anti-hypertensive medication: one using ACE inhibitor combined with hydrochlorothiazide (HCTZ), and one using non-dihydropyridine calcium-channel blocker.	Seven hypertensive (Stage 1 and Stage 2) patients being controlled by changes in lifestyle in association with an anti-hypertensive drug- ACE inhibitor, ARB, BB—either alone or in association with the loop diuretic furosemide.
		Other medications: eight patients using drugs of cardiovascular effects in addition to anti-hypertensive medication: acetyl salicylic acid, anti-coagulants, BBs (carvedilol and metoprolol succinate); one using fibrate, three using spironolactone, and two using amiodarone		Other medications: five patients using Acetyl salicylic acid, anticoagulant drug, spironolactone, and amiodarone
Obesity	22%	29%	0%	7%
	Three patients with obesity Grade 1 and one with Grade 2	Five patients with obesity Grade 1		One patient with obesity Grade 1
Dyslipidemia	22%	35%	27%	27%
		Two patients using statin	One patient using statin	Two patients using statin
Sedentary behavior	22%	41%	33%	27%
Smoking	0%	0%	40%	20%

### Ethics Statement

Written informed consent for this study was obtained from all adult participants and was approved by the Research Ethics Committee of the State University of Rio Grande do Norte (UERN) under protocol number 027.2011 and the Certificate of National System of Ethics in Research (CAEE—SISNEP), protocol number 0021.0.428.000–11. All of the experiments described here were performed according to the human experimental guidelines of the Brazilian Ministry of Health and the Declaration of Helsinki.

### Determination of Death Risk and Stroke Risk

The long-term risk of death over 10 years among patients with chronic Chagas disease is predicted by the presence of the six following characteristics: New York Heart Association/NYHA class III or IV (5 points), cardiomegaly on chest radiograph (5 points), abnormalities of the segmental or global left ventricular echocardiogram (3 points), nonsustained ventricular tachycardia on Holter monitoring (3 points), low-voltage QRS complex on the electrocardiogram (2 points) and male sex (2 points). A risk score derived from the combination of points for each of these characteristics was used to classify the patients as having a low (0–6 points), medium (7–11 points) or high (12–20 points) death risk. The estimated long-term mortality over 10 years in the patients grouped in the low, medium and high death risk groups is 10%, 44%, and 84%, respectively [5].

The stroke risk was based on the presence of systolic dysfunction (2 points) and left ventricular apical aneurysm (1 point), primary alteration of ventricular repolarization on the electrocardiogram (1 point) and age greater than 48 years (1 point). The patients were grouped as having a low (0–2 points), medium (3 points), or high (4–5 points) risk of stroke [50].

### Cytokine and Transcription Factor Expression Levels as Determined by Real-Time PCR

Cytokines (IL-4, IL-9, L-10, IL-17, IL-22, IFN-γ, TGF-β and TNF-α), transcription factors (PU.1, GATA-3, RORγt, AHR, T-Bet, FoxP3) and iNOS mRNA expression levels were determined by real-time PCR (qPCR) of peripheral blood mononuclear cells (PBMCs) isolated from Chagas disease patients. Samples from uninfected healthy individuals were used as controls. Total RNA from the PBMCs was isolated using TRIzol reagent (Invitrogen, Carlsbad, CA, USA) and the SV Total RNA Isolation System (Promega, Madison, WI, USA), and cDNA was synthesized using the ImProm-II Reverse Transcriptase System (Promega). The qPCR was performed using SYBR Green (Invitrogen), and the standard PCR conditions were as follows: 50°C (2 min) and 95°C (10 min) followed by 40 cycles of 94°C (30 s), variable annealing primer temperature (Table 2) (30 s), and 72°C (1 min). The expression mRNA levels of the target genes were determined using the mean Ct values from triplicate measurements to calculate the relative expression levels of the target genes in the chagasic patients compared to those in the healthy subjects and were normalized to the housekeeping gene *β*-*actin* using the 2^–ΔΔCt^ formula.

**Table 2:** The sequences of the primers were designed based on nucleotide sequences in the GenBank Database and were used as follows.

Target	Sense and Antisense sequences	Primer annealing temperature
β-actin	TGACTCAGGATTTAAAAACTGGAA	56.5°C
	GCCACATTGTGAACTTTGGG	
GATA-3	GTCCCTTTCGACTTGCATTT	56.9°C
	TATCCATCGCGTTTAGGCTTC	
T-Bet	AATGCCGAGATTACTCAGCTG	56.9°C
	AAAGTTCTCCCGGAATCCTT	
ROR-γt	TGACCAGATTGTGCTTCTCAAA	58.2°C
	TCCTAACCAGCACCACTTCCAT	
PU.1	AGAAGAAGATCCGCCTGTACCA	60.0°C
	GTGCTTGGACGAGAACTGGAA	
AHR	CAGCGTCAGTTACCTGAGAGCCAAG	65.1°C
	CGCAAACAAAGCCAACTGAGGTGGAAG	
Foxp3	AGGAAAGGAGGATGGACGAA	57.8°C
	AGGCAAGACAGTGGAAACCT	
IL-4	AACAGCCTCACAGAGCAGAAGAC	61.0°C
	GTGTTCTTGGAGGCAGCAAAG	
IL-9	CTTCTGGCCATGGTCCTTAC	59.8°C
	CATGGTCTGGTGCAGTTGTC	
IL-10	AGATCTCCGAGATGCCTTCA	58.8°C
	ATTCTTCACCTGCTCCACGG	
IL-17	CAATGACCTGGAAATACCAA	54.9°C
	TGAAGGCATGTGAAATCGAGA	
IL-22	TTCCAGCAGCCCTATATCACC	60.9°C
	GCTCACTCATACTGACTCCGTG	
IFN-γ	ATGCAGAGCCAAATTGTCTCC	59.0°C
	AGGCAGGACAACCATTACTGG	
TGF-β	ATTGAGGGCTTTCGCCTTAG	58.9°C
	TGTGTTATCCCTGCTGTCACAG	
TNF-α	TTCTGGCTCAAAAAGAGAATTG	55.8°C
	TGGTGGTCTTGTTGCTTAAAG	
iNOS	GTTCTCAAGGCACAGGTCTC	59.1°C
	GCAGGTCACTTATGTCACTTATC	

### Statistical Analysis

Data are reported as the mean ± standard deviation (SD). Comparisons of mRNA expression levels between groups were performed using the Kruskal-Wallis test. In all cases, differences were considered significant when p < 0.05. Spearman’s test was used to determine correlations among the mRNA expression levels of cytokines, transcription factors, iNOS, death risk score and stroke risk score. Our analyses were performed using the PRISM 5.0 (GraphPad, San Diego, CA, USA) statistical program.

## Results

**Fig 1:**
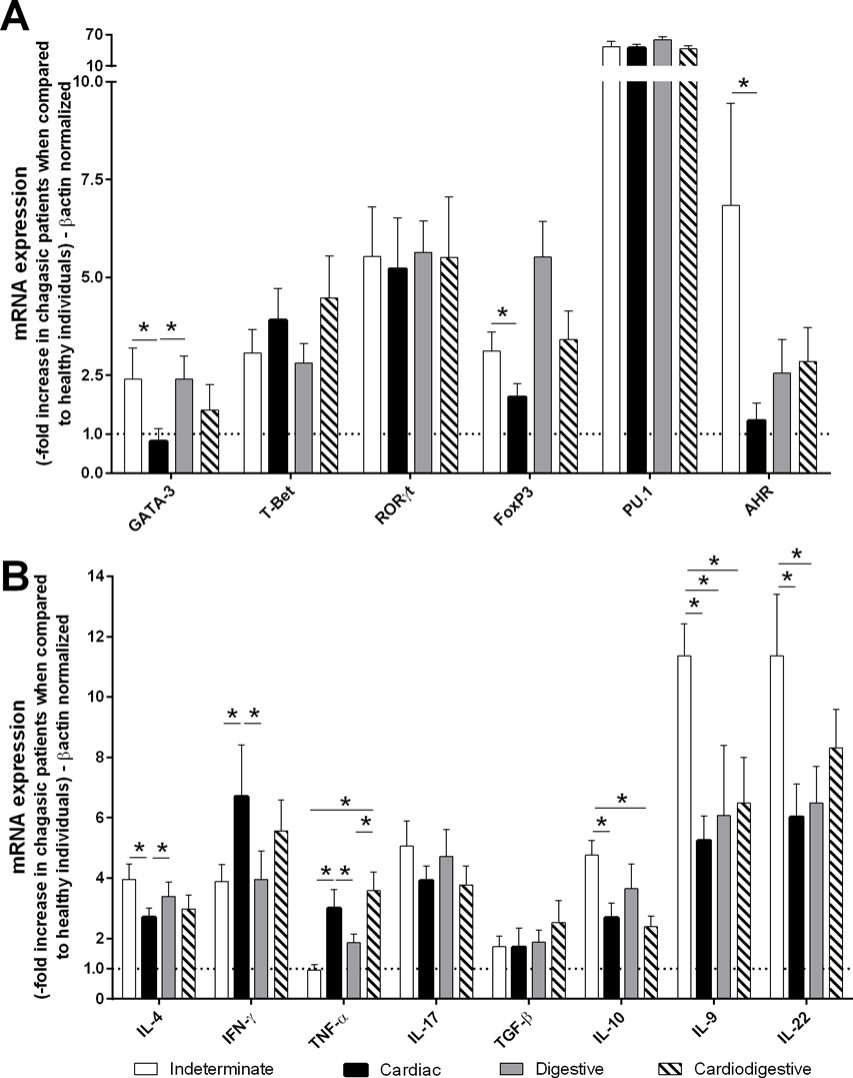
Indeterminate patients exhibited higher GATA-3, Foxp3, AHR, IL-4, IL-9, IL-10, and IL-22 mRNA expression than did cardiac patients. The mRNA expression levels of transcription factors **(A)** and cytokines **(B)** were determined by real-time PCR in peripheral blood mononuclear cells of patients with the indeterminate (n = 18), cardiac (n = 17), digestive (n = 15) and cardiodigestive (n = 15) clinical forms of Chagas disease. The expression levels were normalized to the expression level of *β-actin*. The results are expressed as the means ± standard errors. *P < 0.05.

Initially, we classified the 65 patients according to the clinical form of Chagas disease. The indeterminate, cardiac, digestive and cardiodigestive clinical forms were observed in 27.7% (18/65), 26.1% (17/65), 23.1% (15/65), 23.1% (15/65) of patients, respectively (Table 3). Subsequently, the mRNA expression levels of transcription factors and cytokines mainly expressed in Th1 (T-Bet/IFN-γ and TNF-α), Th2 (GATA-3/IL-4), Th9 (PU.1/IL-9), Th17 (RORγt/IL-17), Th22 (AHR/IL-22) and Treg (Foxp3/IL-10 and TGF-β) were determined in PBMCs by qPCR. Indeterminate patients exhibited higher levels of GATA-3, Foxp3, AHR, IL-4, IL-9, IL-10, and IL-22 mRNA expression than did cardiac patients. However, cardiac patients exhibited higher levels of IFN-γ and TNF-α mRNA compared with indeterminate patients (Fig 1A and 1B).

**Table 3:** Clinical data of chronic chagasic subjects from the Northeast of Brazil included in this investigation.

Clinical involvement	Indeterminate	Cardiac	Digestive	Cardiodigestive
Female Sex–no. (%)	8 (44.5)	6 (35.3)	10 (66.7)	5 (33.3)
Male Sex–no. (%)	10 (55.5)	11 (64.7)	5 (33.3)	10 (66.7)
Total–no. (%)	18 (27.7)	17 (26.1)	15 (23.1)	15 (23.1)
Age–years	41.4 ± 10.7	49.7 ± 11.8	57.6 ± 8.9	65.0 ± 10.6
Megacolon–no. (%)	-	-	9 (60.0)	8 (53.3)
Megaesophagus–no. (%)	-	-	3 (20.0)	3 (20.0)
Megaesophagus and Megacolon–no. (%)	-	-	3 (20.0)	4 (26.7)
Left ventricular ejection fraction ±standard deviation	64.6 ± 3.42	55.8± 14.96	65.0 ± 6.48	56.2±13.84
Cardiothoracic index± standard deviation	0.43 ± 0.05	0.48± 0.05	0.42± 0.03	0.50± 0.05

**Fig 2:**
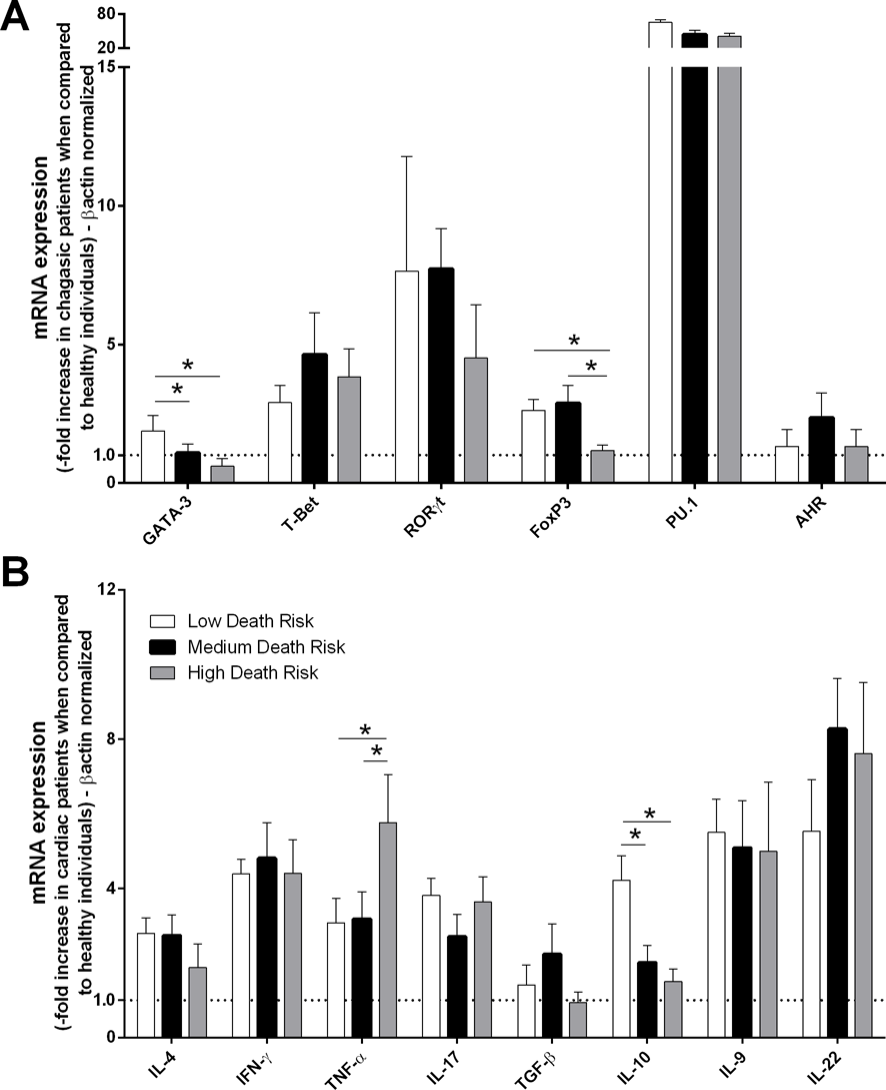
TNF-α, IL-10, T-Bet and FoxP3 expression levels in patients with chronic chagasic cardiomyopathy are correlated with death risk. The mRNA expression levels of transcription factors **(A)** and cytokines **(B)** were determined by real-time PCR in peripheral blood mononuclear cells of patients with the cardiac (n = 17) and cardiodigestive (n = 15) clinical forms of Chagas disease that were classified into high (n = 10), medium (n = 12), and low (n = 10) death risk groups. The expression levels were normalized to the expression level of *β-actin*. The results are expressed as the means ± standard errors. *P < 0.05.

**Fig 3:**
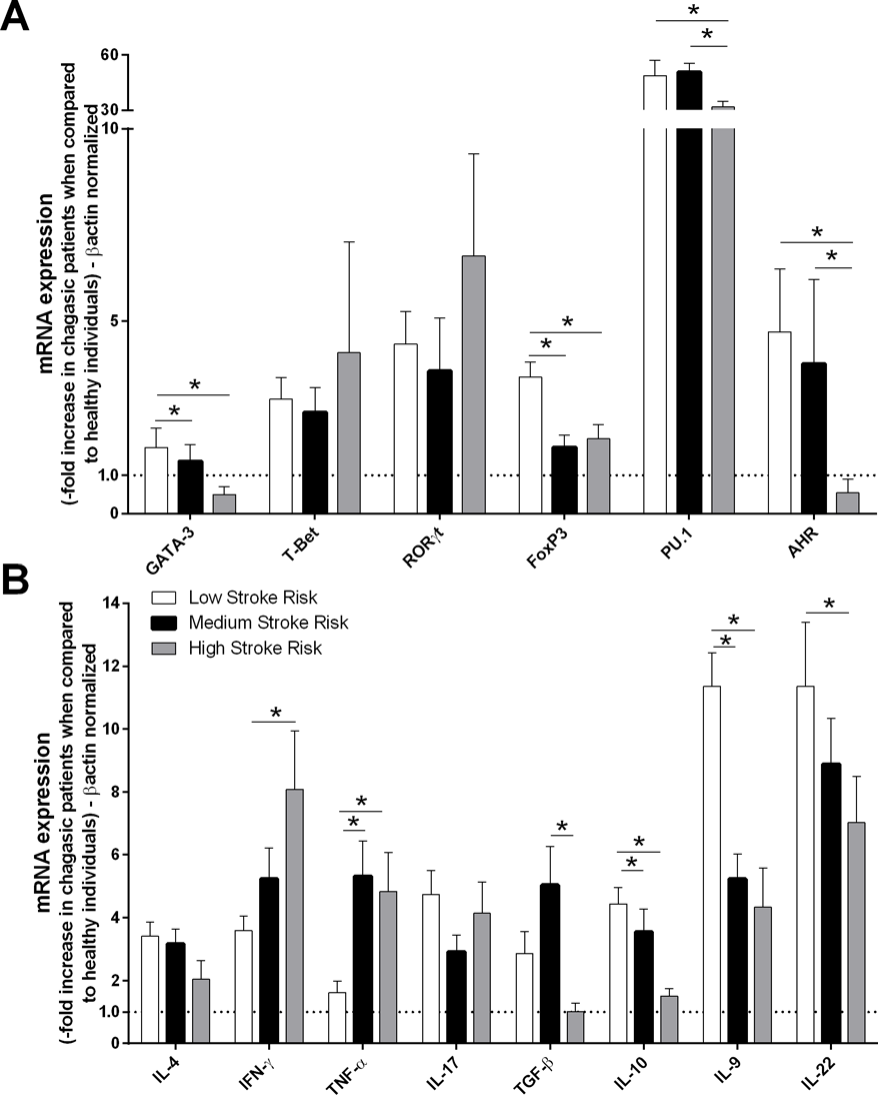
Patients who exhibited low stroke risk also exhibited high GATA-3, Foxp3, PU.1, AHR, IL-9, IL-22 and IL-10 expression. The mRNA expression levels of transcription factors **(A)** and cytokines **(B)** were determined by real-time PCR in peripheral blood mononuclear cells of chagasic patients with low (n = 40), medium (n = 18) and high (n = 7) risks of stroke. The expression levels were normalized to the expression level of *β-actin*. The results are expressed as the means ± standard errors. *P < 0.05.

Patients with chronic chagasic cardiomyopathy (cardiac and cardiodigestive clinical forms) were grouped according to their long-term risk of death over 10 years and were classified as having a low (10/32–31.25%), medium (12/32–37.50%), or high (10/32–31.25%) death risk. The degree of death risk was compared with the production of cytokines and transcription factors. Patients with low death risk exhibited higher expression of FoxP3, GATA-3 and IL-10 than did those with a high death risk (Fig 2A and 2B). Subsequently, patients who exhibited the indeterminate, cardiac, digestive and cardiodigestive clinical forms of Chagas disease were grouped as having a low (40/65–61.54%), medium (18/65–27.69%) or high (7/65–10.77%) stroke risk. The expression levels of cytokines and transcription factors were compared among the patients from different groups. We observed that low stroke risk patients exhibited higher GATA-3, Foxp3, PU.1, AHR, IL-9, IL-10 and IL-22 expression levels than did patients with high stroke risk (Fig 3A and 3B). However, IFN-γ and TNF-α mRNA expression was increased in patients with high stroke risk compared with those with low risk (Fig 3B).

**Fig 4:**
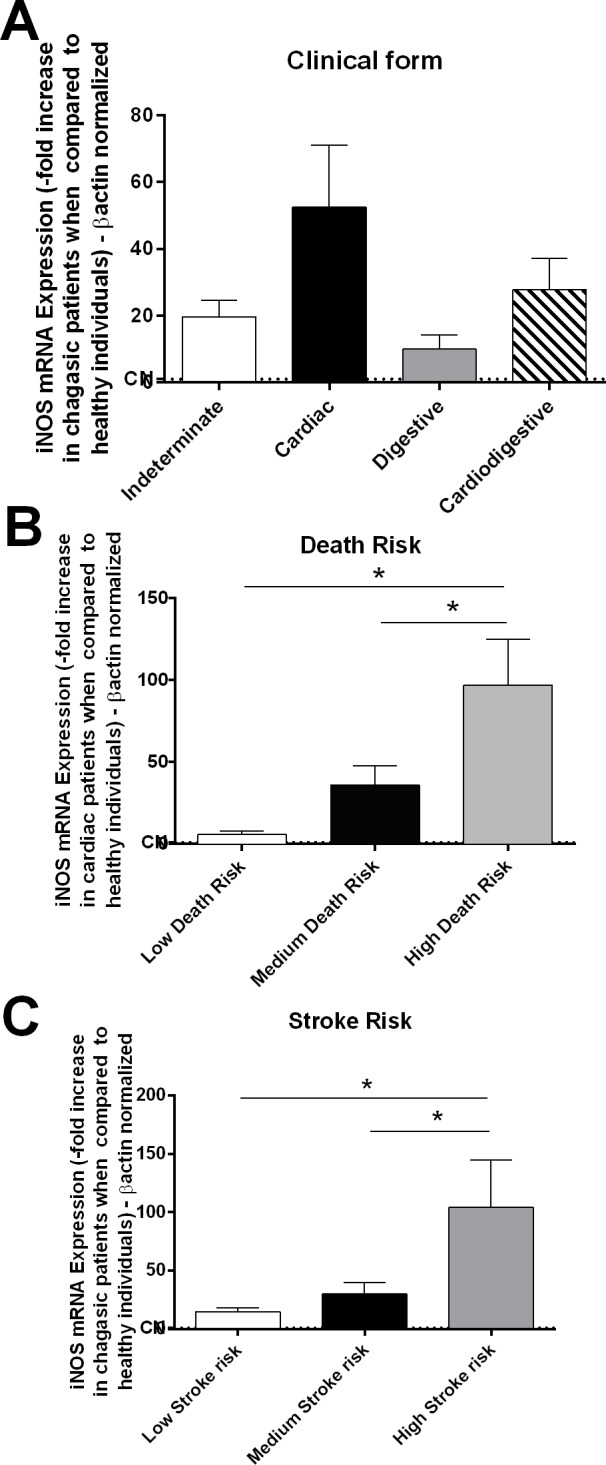
Patients who exhibited high death and stroke risks exhibited high iNOS expression. The mRNA expression levels of iNOS were determined by real-time PCR of peripheral blood mononuclear cells of chagasic patients with the indeterminate (n = 18), cardiac (n = 17), digestive (n = 15) and cardiodigestive (n = 15) forms of disease **(A)**. Patients were classified as having a high (n = 10), medium (n = 12), or low (n = 10) death risk **(B)** and were also grouped as having a low (n = 40), medium (n = 18) or high (n = 7) stroke risk **(C).** The expression of iNOS was normalized to the expression level of *β-actin*. The results are expressed as the means ± standard errors. *P < 0.05.

In an attempt to elucidate the inflammatory mechanism involved in stroke generation, we quantified the mRNA expression of iNOS. Nitric oxide may be involved in the inhibition of endothelial nitric oxide synthase (eNOS), resulting in the vasoconstriction of cerebral arteries. Patients who exhibited different clinical forms of Chagas disease exhibited similar iNOS mRNA levels (Fig 4A). However, those who exhibited high long-term death risk over 10 years and high stroke risk had higher iNOS mRNA expression than those patients with a low or medium risk of death and stroke (Fig 4B and 4C).

**Fig 5:**
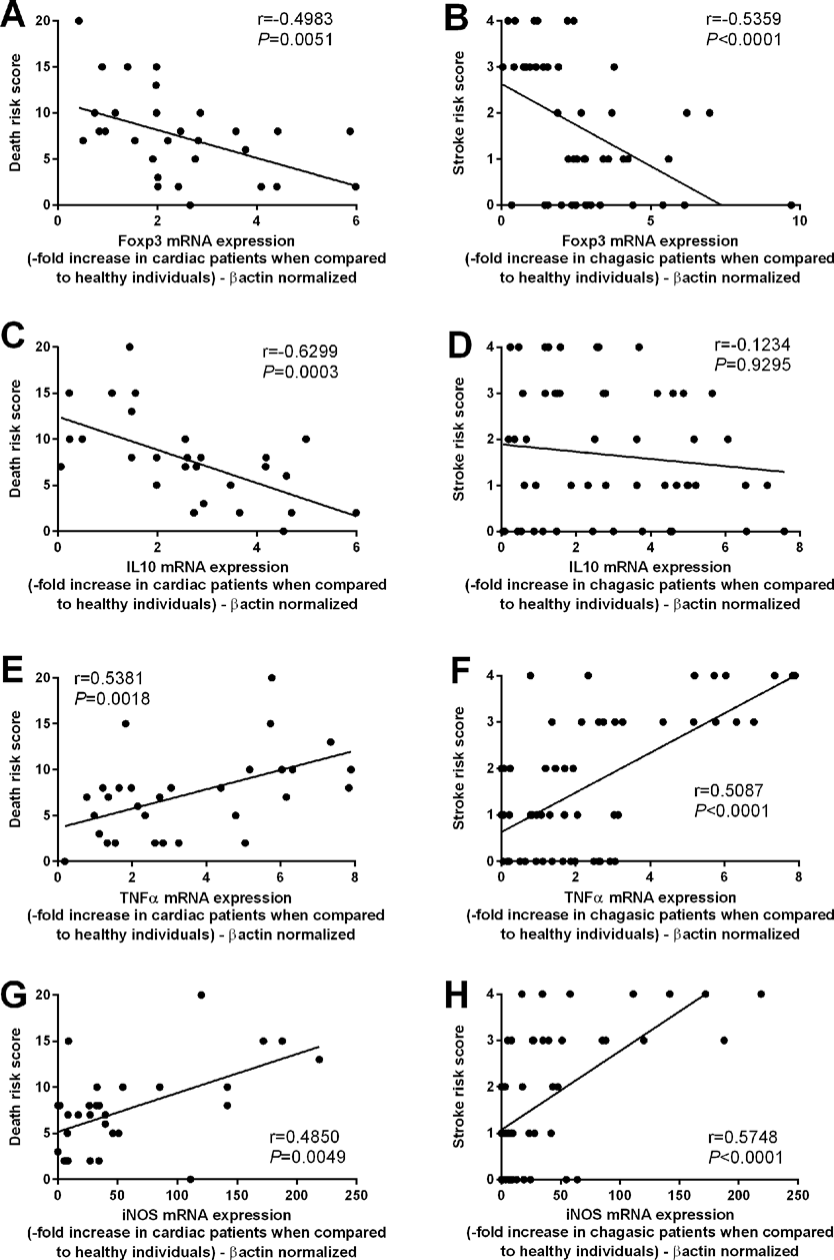
High TNF-α, iNOS and low Foxp3 expression are correlated with the risks of death and stroke. The mRNA expression levels of Foxp3 (**A and B**), IL-10 (**C and D**), TNF-α (**E and F**) and iNOS (**G and H**) were determined by real-time PCR of peripheral blood mononuclear cells from chagasic patients classified as having a high (n = 10), medium (n = 12), or low (n = 10) death risk. The patients were also grouped as having a low (n = 40), medium (n = 18) or high (n = 7) stroke risk. Expression levels were normalized to the expression level of *β-actin*. The results are expressed as the means ± standard errors.

Subsequently, we analyzed the correlation between the mRNA expression of Foxp3, IL-10, TNF-α and iNOS with the death and stroke risks. A negative correlation was observed between Foxp3 and death risk (r = -0.4983; p = 0.0051) (Fig 5A) and stroke risk (r = -0.5359; p < 0.0001) (Fig 5B). Moreover, a negative correlation between IL-10 mRNA expression and death risk (r = -0.6299; p = 0.003) was also observed (Fig 5C). No significant correlation between IL-10 mRNA expression and the stroke risk was observed (r = -0.1401; p = 0.3422) (Fig 5D). A positive correlation was observed between the TNF-α mRNA expression and death risk (r = 0.5381; p = 0.0018) (Fig 5E) and stroke risk (r = 0.5087; p < 0.0001) (Fig 5F); and a positive correlation was also observed between iNOS mRNA expression and death risk (r = 0.4850; p = 0.0049) (Fig 5G) and stroke risk (r = 0.5748; p < 0.0001) (Fig 5H).

## Discussion

To gain a better understanding of the stroke pathobiology in Chagas disease patients, we investigated the correlation of immune mediators with the death and stroke risks in indeterminate, cardiac, digestive and cardiodigestive patients.

We first analyzed the mRNA expression of cytokines (IL-4, IL-9, L-10, IL-17, IL-22, IFN-γ, TNF-α, TGF-β) and transcription factors (PU.1, GATA-3, RORγt, AHR, T-Bet, FoxP3) in PBMCs obtained from Chagas disease patients who exhibited the indeterminate, cardiac, cardiodigestive and digestive clinical forms of the disease. Cardiac patients exhibited higher mRNA expression of IFN-γ, TNF-α and lower mRNA expression of IL-10, Foxp3, AHR, and GATA-3 than those with the indeterminate clinical form of Chagas disease. The immunological imbalance in cardiac patients includes reduced IL-10 production and increases of TNF-α and IFN-γ production [27,51–53]. Resistance to *T*. *cruzi* infection is largely dependent on the production of nitric oxide and its derived nitrogen and oxygen free radicals. The pro-inflammatory cytokines IL-12, IFN-γ and TNF-α (Th1 response-related) activate macrophages to promote parasite killing through the production of trypanocidal radicals [54,55]. In addition, these cytokines also act as a positive feedback for Th1 differentiation. Th1 cells orchestrate an exaggerated CD8^+^ T cell response, causing tissue damage and fibrosis [25]. The regulation of *T*. *cruzi*-induced inflammation occurs primarily through the Th2 and Treg-related cytokines IL-4, IL-10, and TGF-β [27,31,56]. The regulation of inflammation was observed in indeterminate patients, who exhibited high GATA-3 and IL-10 mRNA expression. Biopsies obtained from heart tissue of patients with chronic chagasic cardiomyopathy have showed markedly up regulation of IFN-γ and T-Bet mRNA expression, and lower increases of GATA-3, FoxP3 and CTLA-4 than healthy subjects. Moreover, expression of Th1-related genes such as T-Bet and IFN-γ was correlated with ventricular dilation as well [57]. We also described Th9- and Th22-related mediators and their correlation with clinical forms of Chagas disease. Cardiac patients exhibited lower levels of IL-9, IL-22 and AHR mRNA expression when compared with indeterminate patients. IL-9 also can promote the development of Th17 cells and was reported to be produced by these cells [58]. We have previously demonstrated that indeterminate chagasic patients exhibit increased IL-17 production that can be correlated to the control of cardiac dysfunction [27]. The asymptomatic patients infected with *Leishmania donovani* another trypanosomatid parasite the etiological agent of Kala Azar (KA) produce enhanced amounts of IL-17 maybe contributing to host survival and control of parasite growth [59].

Thus, IL-9 and IL-22 may be involved in regulating the Th1 response and inflammatory cytokine expression in patients with the indeterminate form of the disease, and these cytokines may help prevent the development of chronic chagasic cardiomyopathy.

Subsequently, cardiac patients were categorized in low, medium and high death risk groups [5]. Here, patients with low death risk exhibited increased expression of FoxP3, GATA-3 and IL-10 compared with high death risk patients. Cardiac damage during *T*. *cruzi* infection is due to parasite multiplication and the immune response, both of which destroy cardiac muscle and the autonomous nervous system, causing electrocardiographic changes, cardiomegaly and death [6,60,61]. Patients with indeterminate Chagas disease produce higher levels of IL-10; IL-10 controls the inflammatory immune response generated by the parasitic infection and prevents damage to the myocardium [27]. During the chronic phase of Chagas disease patient mortality is mostly associated with cardiac involvement [3]. Chagasic cardiopathy starts with destruction of myocardial fibers by progressive inflammation with subsequent replacement by fibrotic tissue, an inflammatory and fibrogenic process that ends up in pathologic ventricular remodeling due to a gradual loss of the contractile elements. During remodeling ventricular dysfunction is initially compensatory but the dynamics of the inflammatory process leads to increased cardiac dilatation which evolves to a non-compensatory dilatation, with progressive loss of ventricular ejection capacity. Complex ventricular arrhythmias and failure of mitral- and tricuspid valves further contribute to the worsening of the cardiopathy and might be an additional risk factor within the pleiad of mortality-related mechanisms [61,62]. The fibrosing and progressive chronic myocarditis is also the key substrate for impairment of the conduction system in Chagas disease [62]. Macrophages, T lymphocytes (CD4^+^ and CD8^+^), cytokines and autoantibodies associated with the presence of the parasite and/or their antigens participate in myocardial lesion formation [27,46,63–65]. Inflammatory cytokines (TNFα and IFNγ) have been found in myocardial biopsies of chagasic patients [51] in association with parasitism and inflammation, a suggestive evidence for their possible relationship with neuronal depopulation [66]. Direct ganglionar parasitism is found associated with periganglionitis, and nervous fiber- and Schwann cell degenerative lesions. Direct parasitism is observed, as well as nervous fiber- and degenerative lesions [67]. Deposition of autoantibodies in structures of the neurotransmitter receptors (β-adrenergic receptors, muscarinic receptors) might cause desensitization resulting in progressive denervation, an event that may also be implicated in the occurrence of ventricular arrhythmias [66]. Antibodies from patients with chronic Chagas disease displaying complex arrhythmias decrease the heart rate and cause atrioventricular block in isolated rabbit hearts [68,69], indicating that the immune response is an important pathophysiological factor in the development of complex arrhythmias and cardiac death in Chagas disease [70]. Despite limitations, experimental and clinical studies strongly support the notion that functional and structural microvascular abnormalities occur in Chagas cardiomyopathy, possibly as a consequence of the underlying inflammatory process [62]. Actually, as argued by Kania and co-workers [71] recent findings suggest that heart-infiltrating monocyte-like cells indeed contain a pool of progenitors, which represent the cellular source both for accumulation of differentiated monocytes during the acute inflammatory phase and for transforming growth factor-β-mediated myocardial fibrosis during the later chronic stages of disease. Obviously, a delicate balance of proinflammatory and profibrotic cytokines dictates the fate of bone marrow-derived heart-infiltrating progenitors and directly influences the morphologic phenotype of the affected heart. Given the magnitude of the question of sudden death in chronic Chagas disease patients and high cost of medical treatment, identifying the patient at risk and outlining the process that initiated or facilitated these arrhythmias is a high priority issue in such a way that those patients might be more effectively treated.

Infectious and parasitic diseases contribute to stroke risk [72]. It has been previously shown that chagasic patients have an increased risk of stroke, independent of cardiac function (LVEF) [5,73]. In this study, we demonstrated that patients with low stroke risk have increased mRNA expression of GATA-3, Foxp3, PU.1, AHR, IL-9, IL-22 and IL-10. These mediators can regulate the inflammatory response (TNF-α and IFN-γ) associated with the mechanism of thrombus formation. Also, we observed that high stroke risk patients exhibited high mRNA expression of IFN-γ. Patients with Chagas disease produce inflammatory mediators that increase the chance of thromboembolic phenomena [74]. The cytokine IFN-γ induces TNF-α production and causes increased expression of ICAM-I (intracellular adhesion molecule-I) and VCAM-I (intravascular adhesion molecule-I), both of which are involved in the cell adhesion process and surface formation of thrombi [20,75]. TNF-α also modulates endothelial cell coagulant properties, markedly increasing tissue factor-like procoagulant activity in cultured human endothelial cells [76]; TNF-α also stimulates increased cellular surface adhesivity in polymorphonuclear leukocytes, monocytes, lymphocytes and leukocyte cell lines [77,78]. The classic elements of the thrombus formation, such as endothelial damage, decreased blood flow and imbalance between coagulation factors, are increased in patients with Chagas disease. These elements are altered primarily by the inflammatory response generated against the parasite [61,74].

The inflammatory response to the parasite could affect the vasodilatation of the cerebral arteries, thus contributing to stroke formation. Nitric oxide produced by eNOS activates guanylate cyclase in vascular smooth muscle cells by increasing cGMP levels causing vasodilatation [79]. After *T*. *cruzi* infection there is macrophage activation with iNOS production and these cells invade endothelium and migrate to tissues. High nitric oxide production in the vascular endothelium of chagasic patients due to high iNOS activation could lead to eNOS inhibition, vasoconstriction and cerebral microvascular spasms, causing ischemic stroke [80]. In this study, patients who exhibited high long-term death risk over 10 years and patients with a high stroke risk exhibited higher iNOS mRNA expression than those patients with low risk of stroke and death. Moreover, a positive correlation was observed between iNOS expression and death and stroke risk. The nitric oxide produced by iNOS inhibits eNOS [80].

Our findings suggest that chagasic patients with high stroke and death risks exhibit reduced expression of cytokines related to Th2, Th9, Th22 and Treg profiles. The decreased production of these cytokines may be correlated to increased vascular inflammatory processes that subsequently lead to thrombi and atherosclerosis formation. Patients with high risks of stroke and death exhibited high iNOS mRNA expression, indicating that the patients likely had increased nitric oxide production in the vascular endothelium. The high levels of nitric oxide likely could led to eNOS inhibition and vasoconstriction, thus contributing to the stroke pathophysiology. Moreover, key cytokines of the Th2, Th9, Th22 and Treg profiles are correlated with the indeterminate clinical form of Chagas disease. The present study unveiled the existence of an immunopathological outcome underlying chagasic patients condition that involves an imbalanced expression of IL-10, FoxP3 and iNOS, which increases the risk of stroke or death. An improved understanding of the immunological mechanisms involved in ischemic strokes in Chagas disease patients may also contribute to the reduction of stroke-related mortality and morbidity in the general population and may lead to the development of prophylactic or therapeutic therapies.
